# Evaluation of banana germplasm and genetic analysis of an F_1_ population for resistance to *Fusarium oxysporum* f. sp. *cubense* race 1

**DOI:** 10.1007/s10681-019-2493-3

**Published:** 2019-09-23

**Authors:** Ivan Kabiita Arinaitwe, Chee How Teo, Fatimah Kayat, Robooni Tumuhimbise, Brigitte Uwimana, Jerome Kubiriba, Rony Swennen, Jennifer Ann Harikrishna, Rofina Yasmin Othman

**Affiliations:** 1 Institute of Biological Sciences (Genetics), Faculty of Sciences, University of Malaya, Kuala Lumpur, Malaysia; 2 National Banana Research Program, National Agricultural Research Organization, Kampala, Uganda; 3 Universiti Malaysia Kelantan, Jeli, Malaysia; 4 Centre for Research in Biotechnology for Agriculture (CEBAR), University of Malaya, Kuala Lumpur, Malaysia; 5 International Institute of Tropical Agriculture (IITA), Kampala, Uganda; 6 International Institute of Tropical Agriculture (IITA), POB 10, Duluti, Arusha, Tanzania

**Keywords:** *Musa* spp., Fusarium wilt, Inheritance, Segregating population, Dominant genes, *Foc*

## Abstract

Fusarium wilt of bananas (*Musa* spp.), caused by *Fusarium oxysporum* f. sp. *cubense* (*Foc*) causes up to 100% yield loss in bananas. *Foc* race 1 in particular is very devastating to dessert bananas in Uganda. One of the effective control strategies for the disease is the development of resistant cultivars through breeding. The objectives of this study were to identify suitable banana germplasm for generating a segregating population for resistance to *Foc* race 1 and understand the mode of inheritance of resistance to *Foc* race 1. Twenty-two banana accessions sourced from the National Agricultural Research Organisation in Uganda were challenged with *Foc* race 1 in a screen house experiment. Monyet, resistant to *Foc* race 1 and Kokopo, susceptible, were selected and crossed to generate 142 F_1_ genotypes. These F_1_ genotypes were also challenged with *Foc* race 1 in a screen house experiment. Data were collected on rhizome discoloration index (RDI), leaf symptom index (LSI) and pseudo-stem splitting (PSS), and analysed for variability. The banana accessions evaluated showed varying degrees of resistance to *Foc* race 1. Segregation ratios for resistant versus susceptible progenies fitted 13:3 (χ^2^ = 0.12, *P* = 0.73) for RDI and 11:5 (χ^2^ = 3.04, *P* = 0.08) for PSS. Estimated broad sense heritability was 27.8% for RDI, 13.9% for LSI and 14.7% for PSS. The results suggest that resistance to *Foc* race 1 in banana is controlled by at least two dominant genes with epistatic interaction and that heritability of resistance to *Foc* race 1 is low in *Musa* spp.

## Introduction

Banana (*Musa* spp.) a heterogeneous, outcrossing and vegetatively propagated crop (Ortiz and Swennen 2014), is cultivated in more than 130 countries in the world (FAOSTAT 2016). Its total production worldwide is estimated at 162 Mio. Metric Tons (MMT), with 21 MMT (14%) deemed for export, earning about US$100 billion (FAOSTAT 2016). Although industrialised nations view banana essentially as a dessert item, many regions of the developing world consider bananas as an essential staple that contributes significantly to the caloric intake of low-income subsistence farmers (Etebu and Young-Harry 2011; Brown et al. 2017). It is a food and cash crop for more than 70 million smallholder farmers in the Great Lakes Region of Africa, with an annual production worth US$ 4.3 billion, which is about 5% of the region’s gross domestic product (EAC 2012).

Uganda is the largest producer of bananas in Africa with an annual total production of 10 MMT (Kilimo-Trust 2012). Most of the bananas grown in the country are the locally evolved clones known as the East African highland bananas (EAHBs, denoted *Musa* AAA-EA). The EAHBs include cooking ‘Matooke’ and brewing ‘Mbidde’ types, representing 70% and 20% of the total bananas produced, respectively. The rest of the bananas grown are dessert types, that include ‘Gros Michel' (syn. ‘Bogoya', AAA), ‘Pisang Awak’ (syn. ‘Kayinja', ABB) and Ney Poovan (‘Sukali Ndiizi', AAB and ‘Kisubi’, AB), constituting about 9%, and the plantains (AAB), constituting 1% (Karamura and Karamura 1994; Gold et al. 2002). Dessert bananas are widely eaten when ripe and used in local beer breweries (Van Asten et al. 2010; Karangwa et al. 2016).

Fusarium wilt, also known as Panama disease, is the most important lethal disease of dessert bananas (Bidabadi and Sijun 2018). It is a soil-borne fungal disease caused by *Fusarium oxysporum* f. sp. *cubense* (*Foc*) (Ploetz 2015a, b). *Fusarium oxysporum* f. sp. *cubense* race 1 is the primary cause of Fusarium wilt disease of dessert bananas in Uganda (Karangwa et al. 2016). *Foc* race 1 is reported to cause an estimated yield loss of > 60% in dessert bananas (Tushemereirwe et al. 2000). Controlling Fusarium wilt using chemical, biological and cultural control methods has not been very effective (Guo et al. 2013) partly due to long-term survival of the spores in soil and due to the ability to evolve into new strains able to infect resistant cultivars (Su et al. 1986; Mostert et al. 2017). Cultural practices such as pruning symptomatic leaves, culling and burying of diseased plants have been applied for the control of Fusarium wilt, however, these practices lead to further propagation of the disease as the spores can survive in soil for long periods, with or without an alternate host. Chemical control methods are also hazardous to the environment, domestic animals and humans (Ploetz 2000; Pérez-vicente et al. 2014; Ploetz 2015a, b).

Host plant resistance is an effective alternative to chemical, cultural and biological methods for controlling diseases in banana such as Fusarium wilt (Ploetz 2000). It is durable, environmentally safe and user-friendly for small-scale farmers. Natural sources of fungal and other disease resistance exist in wild species and in synthetic diploids of banana developed by breeding programmes (Uma et al. 2011; MusaNet 2016). These diploids have been used in disease resistance in introgressive hybridization programmes (Tushemereirwe et al. 2014; Brown et al. 2017). Conventional banana breeding is highly challenging due to several factors including a long-life cycle, leading to a long breeding cycle (Popova 2011; Brown et al. 2017) and due to the large space requirement, resulting in high costs. The polyploid nature and low female fertility of most popular cultivars of banana (Nyine et al. 2018) and limited knowledge on the genetics of resistance to pests and diseases, have also significantly hindered banana breeding (Heslop-Harrison and Schwarzacher 2007). The success of genetic resistance breeding strategies is affected by the number of genes involved and the nature of inheritance (Boerma and Hussey 1992; Mundt 2014). Therefore, identifying the sources of resistance and studying the genetics underlying resistance to *Foc* race 1 is pertinent to support banana breeding programmes. The objectives of the present study were: (1) to identify suitable banana germplasm to utilise in generating a segregating banana population for resistance to *Foc* race 1 and (2) to understand the mode of inheritance of resistance to *Foc* race 1.

## Materials and methods

Plant germplasm used in the development of segregating population for Fusarium wilt resistance.

A total of 22 parental banana germplasm comprised of 18 diploids, one tetraploid and three triploids (Table 1) was used. The germplasm was sourced from the National Agricultural Research Organisation (NARO) and International Institute of Tropical Agriculture (IITA) in Uganda. Triploid bananas were used as controls. Selection of the germplasm was based on good agronomic traits and varying degrees of resistance to several pests and diseases of economic importance in Uganda.

**Table 1:** Characteristics of Banana germplasm challenged with *Foc* race 1

Accession number	Germplasm	Ploidy	Source	Resistance to *Foc* race 1	Other attributes
–	TMB2X614-1	2x	IITA-Uganda	Unknown	–
ITC1511	Pahang	2x	IITA-Uganda	Unknown	–
ITC1243	Kokopo	2x	IITA-Uganda	Unknown	Source of Vitamin A (Orange-fleshed)
ITC0093	Long Tavoy	2x	IITA-Uganda	Unknown	–
1TC0966	Zebrina GF	2x	IITA-Uganda	Unknown	Dwarf stature, big finger size
ITC0591	Kasaska	2x	NARO-Uganda	Unknown	Susceptible to banana weevil, Source of Vitamin A (Orange-fleshed), Big finger size
ITC0253	Borneo	2x	NARO-Uganda	Unknown	Resistant to banana weevil
ITC1121	Pisang Lilin	2x	IITA-Uganda	Unknown	–
ITC1179	Monyet	4x	IITA-Uganda	Unknown	–
MMC453	Mwitu Pemba	2x	NARO-Uganda	Unknown	–
MMC486	Hutishamba	2x	NARO-Uganda	Unknown	Edible, Susceptible to black Sigatoka
ITC 1468	Kahuti	2x	NARO-Uganda	Unknown	Edible, Susceptible to black Sigatoka
MMC453	Mlelembo	2x	NARO-Uganda	Unknown	Edible, Susceptible to black Sigatoka
MMC419	Mraru	2x	NARO-Uganda	Unknown	Edible, Susceptible to black Sigatoka
ITC1466	Nshonowa	2x	NARO-Uganda	Unknown	Edible, Susceptible to black Sigatoka
MMC418	Njuru	2x	NARO-Uganda	Unknown	Edible, Susceptible to black Sigatoka
MMC248	TMB2X8075-7	2x	NARO-Uganda	Resistant	Nematode resistance (*Radopholus similis*)
MMC501	Mshale	2x	NARO-Uganda	Unknown	Edible, Susceptible to black Sigatoka
ITC0249	Calcutta 4	2x	NARO-Uganda	Resistant	Resistant to banana weevil, nematodes, black Sigatoka and male and female fertile
MMC176	Kayinja	3x	NARO-Uganda	Susceptible control	Edible desert
MMC167	Sukali Ndiizi	3x	NARO-Uganda	Susceptible control	Edible desert
MMC021	Mbwazirume	3x	NARO-Uganda	Resistant control	Edible triploid

### Experimental site

The experiments for challenging the collected parental banana germplasm and F_1_ population with *Foc* race 1 inoculum were conducted in a screenhouse at the National Agricultural Research Laboratories (NARL), Kawanda from April 2015 to June 2018. The National Agricultural Research Laboratories are located in Central Uganda at 32°36′E and 0°25′N, 1210 m above sea level. Kawanda is a hotspot of many pathogens and pests, including *Mycosphaerella fijiensis ‘*Morelet’, *Foc* race 1, weevils and nematodes.

### Preparation of *Foc* race 1 inoculum

*Foc* race 1, VCG 0124 inoculum was prepared following a protocol described by Ssali et al. (2013) with some modifications. The *Foc* fungus was isolated by culturing corm pieces (1cm^3^) from suckers of symptomatic ‘Sukali Ndiizi’ that was collected from a “hotspot” previously diagnosed with *Foc* race 1, VCG 0124 infection at NARL, Kawanda, in Uganda. The corm pieces were sterilised by soaking them in 15% Sodium hypochlorite and then in 70% ethanol for 15 min at each soaking stage. The corm pieces were rinsed four times in sterile water and plated onto potato dextrose agar media (PDA) supplemented with streptomycin (300 μg/mL). The *Foc* cultures were incubated in the dark at 25 °C with routine subculturing until pure cultures with purplish-whitish mycelia were achieved. A PDA plate (90 × 15 mm) fully colonised by pure *Foc* mycelium, was inoculated into 1 kg of sterile millet grains that had been autoclaved at 121 °C for 30 min then cooled before inoculation. The inoculated millet substrate was incubated for 10 days at 25 °C in the dark with daily agitation to ensure uniform fungal growth.

### *Foc race* 1 disease challenge assay

Three-month-old tissue-cultured banana plants were planted into plastic polythene pots containing 10 kg of sterile loam soil mixed with 100 g of millet grains colonized with *Foc* race 1. The experiments were set up in a randomised complete block design (RCBD) with six replications. After planting, experimental plants were maintained in a screen house at 28 °C for 12 h of daylight for 60 days**.** Fusarium wilt disease was assessed on each plant using the severity level of the three key disease symptoms namely, rhizome discoloration index (RDI), leaf symptom index (LSI) and pseudo-stem splitting (PSS) as described by Viljoen et al. (2017) (Table 2). Specifically, LSI data was recorded at 14 days after inoculation by scoring the yellowing of leaves, while data on PSS and RDI were recorded at 60 days after inoculation.

**Table 2:** Scale for scoring different parameters for Fusarium wilt resistance (Viljoen et al. 2017)

Disease rating scale	Leaf symptom index (LSI)	Pseudo-stem splitting (PSS)	Rhizome discoloration index (RDI)
1	No yellowing	No cracking	No internal symptoms
2	Yellowing of < 1/3 of the leaves	Slight cracking	Few internal spots
3	Yellowing of 1/3 to 2/3 of leaves	Advanced	< 1/3 discolored
4	Yellowing of > 2/3 of the leaves		1/3–2/3 Discoloured
5	Plant dead		> 1/3 Discoloured
6	–		Entire inner rhizome

### Developing an F_1_ population segregating for Fusarium wilt resistance

Monyet (*Musa acuminata* subsp Zebrina), a resistant parental genotype to *Foc* race 1 VCG 0124 was crossed with Kokopo (*Musa acuminata* subsp Banksii), a susceptible parental genotype, to generate 142 F_1_ progenies. Controlled hand pollinations were conducted as described by Ortiz and Vuylsteke (1995). Embryos of the F_1_ hybrid seeds were extracted for culture as described by Vuylsteke et al. (1990). The ploidy level of the progenies was determined by flow cytometry method as described by Doležel and Bartoš (2005).

### Data analysis

In order to select two contrasting parents for Fusarium wilt resistance to be used in generating segregating progenies for Fusarium wilt resistance, the data collected from the 22 banana accessions assessed for resistance to *Foc* race 1 were subjected to analysis of variance (ANOVA) using GenStat (Payne et al. 2011). Means of RDI, PSS, and LSI were separated using least significance differences (LSD) at 5% significance level. The Disease Severity Index (DSI) of each genotype was computed for RDI as described by Mak et al. (2004) and the germplasm were placed into respective resistance groups following the method described by Sutanto et al. (2013) (Table 3). The genetic basis underlying Fusarium wilt resistance in F1 progeny was determined from the observed frequencies using the Chi-square test (Cochran 1952) versus the standard genetic ratios (Laughlin 1918; Mendel 1866). To determine the broad sense heritabilities (H), the genotypic (σ^2^g), phenotypic (σ^2^p) and error (σ^2^e) variances were computed using the formulae of Burton and DeVane (1953) and Kebere et al. (2006) as σ^2^g = (MSg−MSe)/r; σ^2^p = σ^2^g + σ^2^e and σ^2^e = MSe, where MSg = genotypic mean square, MSe = environmental variance (error mean square) and r = the number of replications. Heritability was estimated by the formulae of Wricke and Weber (1986): H = σ^2^g / σ^2^p.

**Table 3:** Translation of DSI for LSI and RDI into resistance groups

DSI (RDI)	DSI (LSI)	Translation
1	1	Highly resistant
1.1–3.0	1.1–2.0	Resistant
3.1–5.0	2.1–3.0	Susceptible
5.1–6.0	3.1–5.0	Highly susceptible

*DSI* disease severity index, *RDI* rhizome discoloration index, *LSI* leaf symptom index

## Results

### Variation of the parental banana germplasm for Fusarium wilt

Genotype mean squares determined by ANOVA were highly significant (*P* < 0.001) for RDI and LSI as the measure of Fusarium wilt resistance and non-significant for PSS (*P* > 0.05) (Table 4).

**Table 4:** Analysis of variance of rhizome discoloration index (RDI) and leaf symptom index (LSI) of 22 banana parental germplasm evaluated for *Foc* race 1 in Uganda

Source of variation	df	Mean squares		
		RDI	LSI	PSS
Replication	5	2.2	0.17	0.01
Genotype	21	11.90***	1.61***	0.01^ns^
Residual	105	1.48	0.37	0.01

* ns * non-significant at 0.05 probability level, *df* degrees of freedom

***Significant at 0.001 probability level

### Mean performance of the genotypes for rhizome discoloration index and leaf severity index

**Fig. 1:**
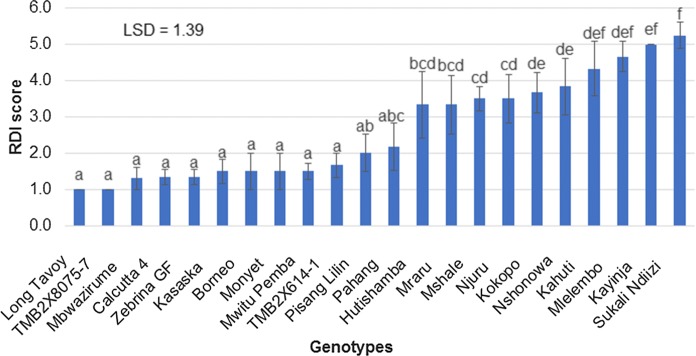
Mean rhizome discoloration index (RDI) comparison among banana accessions challenged with *Foc* race 1 (error bars represent standard error, n = 6, letters represent LSD for mean separation)

**Fig. 2:**
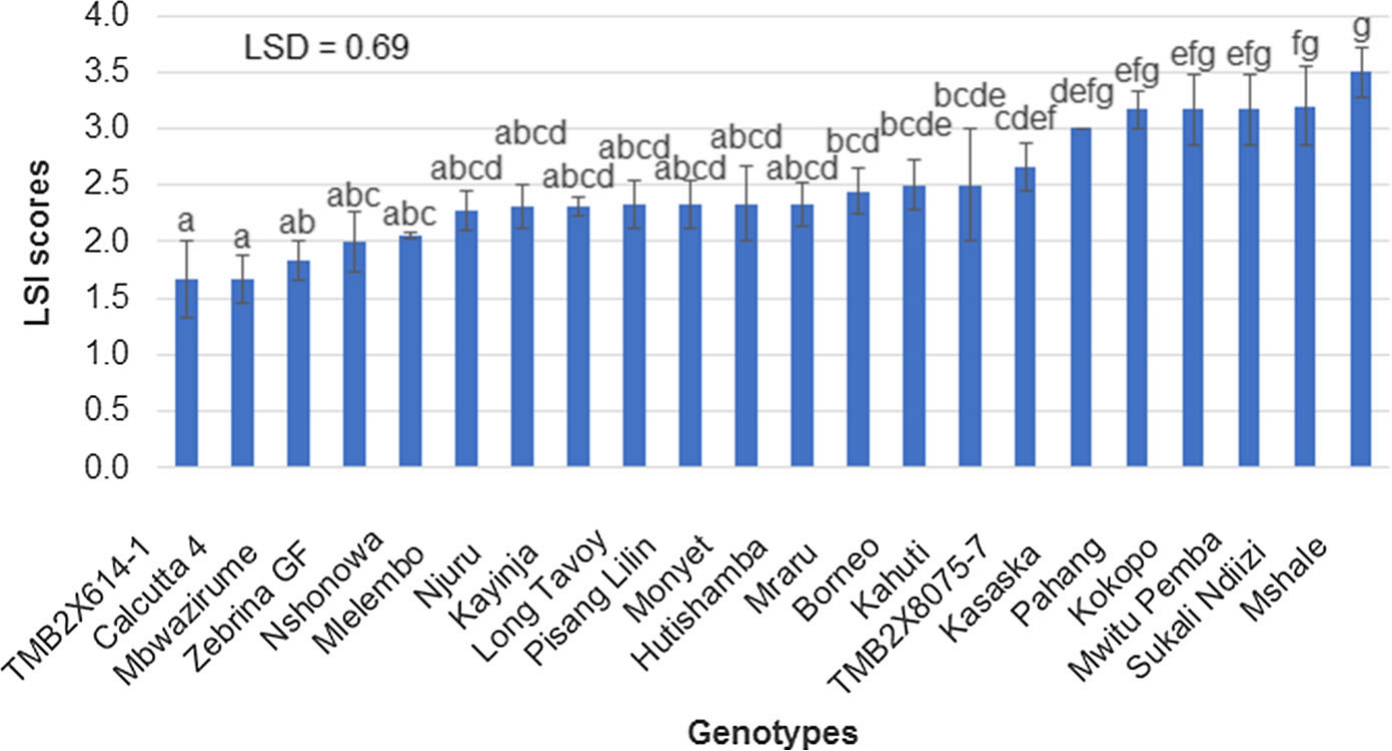
Mean leaf symptom index (LSI) comparison among banana accessions challenged with *Foc* race 1 (error bars represent standard error, n = 6, letters represent LSD for mean separation)

Genotypes Hutishamba, Mraru, Mshale, Njuru, Nshonowa, Kahuti, Mlelembo which belong to the AA-Mchare group, and Kokopo, which originates from Papua New Guinea, were significantly different from the resistant control (Mbwazirume) according to the standard error mean separations and LSD between means of the genotypes for RDI (Fig. 1). The remaining genotypes were not significantly different from resistant control (Mbwazirume). Long Tavoy and TMB2X8075-7 showed a slightly lower RDI mean score than the resistant control ‘Mbwazirume’. The standard error means separation and LSD between the means of the genotypes for LSI could not significantly differentiate the resistant control ‘Mbwazirume’ from the susceptible control ‘Kayinja’ (Fig. 2). Furthermore, some genotypes such as TMB2X8075-7, Kasaska, Borneo, and Mwitu Pemba showed high LSI values (Fig. 2) although they had lower RDI values (non-significant RDI values compared to resistant control Mbwazirume).

### Grouping of parental germplasm into resistance groups using DSI for RDI

Since the standard error means separation and LSD between means of the accessions for the external symptoms/LSI could not significantly differentiate the resistant control ‘Mbwazirume’ from the susceptible control ‘Kayinja’, grouping the genotypes into resistance groups was performed only for values of DSI for RDI. Therefore, based on DSI for RDI values, genotypes were grouped into four resistance classes: highly resistant, resistant, susceptible and highly susceptible. Only 2 genotypes were grouped as highly resistant, 10 as resistant, 8 as susceptible and 2 as highly susceptible (Table 5).

**Table 5:** A categorisation of banana germplasm into *Foc* race 1 resistance groups using DSI for RDI

Highly resistant (DSI = 1.0)	Resistant (DSI = 1.1–3.0)	Susceptible (DSI = 3.1–5.0)	Highly Susceptible (DSI = 5.1–6.0)
Long Tavoy	Mbwazirume (control)	Hutishamba	Kayinja (control)
TMB2X8075-7	Calcutta 4	Mraru	Sukali Ndiizi (control)
	Zebrina GF	Mshale	
	Borneo	Njuru	
	Kasaska	Kokopo	
	Monyet	Nshonowa	
	Mwitu Pemba	Kahuti	
	TMB2X614-1	Mlelembo	
	Pisang Lilin		
	Pahang		

### Genetic basis of the banana resistance to Fusarium wilt

A cross combination of Monyet (tetraploid) and Kokopo (diploid) resulted in 142 F_1_ progenies with a mixture of ploidy levels: 136 triploids (3x), 4 tetraploids (4x) and 2 diploids (2x). The genotype mean squares in the ANOVA for 142 F_1_ progenies were highly significant (*P* < 0.001) for RDI, LSI and PSS (Table 6).

**Table 6:** Analysis of variance of rhizome discoloration index (RDI), leaf severity index (LSI) and pseudo-stem splitting (PSS) of F_1_ progenies from a cross of Monyet and Kokopo

Source of variation	df	Mean squares		
		RDI	LSI	PSS
Replication	5	2.36	0.42	0.32
Genotype	141	5.66***	0.81***	0.34***
Residual	705	1.71	0.41	0.17

*df* degrees of freedom

***Significant at 0.001 probability level

Using DSI for RDI, 117 F_1_ progenies were grouped as resistant (scale of 1.0–3.0) and 25 F_1_ progenies as susceptible (Scale 3.1–6.0) while using DSI for LSI, 73 F_1_ progenies were grouped as resistant (Scale 1.0–2.0) and 69 F1 progenies as susceptible (Scale 2.1–5.0). Using DSI for PSS, 88 F_1_ progenies were grouped as resistant (Scale 1.0) and 54 as susceptible (1.1–3.0). The segregation ratio for RDI fitted the two gene model ratio of 13:3 while PSS fitted the two gene model ratio of 11:5 using a chi square goodness of fit test (Table 7). LSI segregation did not fit either of the one gene model ratios nor the two gene model ratios tested. The 13:3 ratio is described as complete dominance at both gene pairs; however, when either gene is dominant, it overshadows the effects of the other gene, while a ratio of 11:5 indicates complete dominance for both gene pairs only if both kinds of dominant alleles are present; otherwise, the recessive phenotype appears.

**Table 7:** The goodness of fit χ^2^ test for the response of 142 F_1_ banana progenies from Monyet x Kokopo following inoculation with *Fusarium oxysporum* f. sp. *cubense* race 1

Parameter	Genetic ratio	Resistant	Susceptible	χ^2^	χ^2^ (Probability)
RDI	13:3	117	25	0.12	0.73
PSS	11:5	88	54	3.04	0.08

χ^2^ Chi-square test statistic

Estimates of broad-sense heritability for the resistance parameters RDI, LSI and PSS as measures of Fusarium wilt were relatively low (Table 8). RDI had a heritability of 27.8%, while LSI and PSS had heritabilities of 13.9% and 14.7%, respectively.

**Table 8:** The estimated heritability of resistance to *Fusarium oxysporum* f*.* sp*. cubense* race 1 traits in the F_1_ population

Source of variation	df	Mean squares		
		RDI	LSI	PSS
Replication	5	2.4	0.4	0.3
Genotype	141	5.7	0.8	0.4
Residual	705	1.7	0.4	0.2
VE		1.7	0.4	0.2
VG		0.7	0.1	0.03
VP		2.4	0.5	0.2
Heritability (%)		27.8	13.9	14.7

*VE* error variance, *VG* genotypic variance, *VP* phenotypic variance, *df* degrees of freedom

## Discussion

Fusarium wilt [*Fusarium oxysporum* f. sp. *cubense* (*Foc*)] is a destructive soil-borne fungal disease that causes heavy yield losses among susceptible bananas worldwide. *Foc* race 1 was responsible for the destruction of 40,000 hectares of Gros Michel plantations in the Central American/Caribbean region in 1940 (Ploetz and Pegg 2000). In Uganda, *Foc* race 1 heavily affects the dessert banana cultivars, leading to complete destruction of the fields if not controlled (Tushemereirwe et al. 2004). Fusarium wilt can be appropriately addressed by providing farmers with resistant varieties through breeding. Therefore, identifying sources of resistance to *Foc* race 1 and understanding genetic mechanisms underlying *Foc* race 1 resistance are a fundamental step towards breeding resistant banana varieties.

The first part of this study included an assessment of 22 parental banana accessions that had never been utilised for *Foc* race 1 resistance breeding because their response to *Foc* race 1 was unknown. However, upon assessment of the parental genotypes, the mean squares in the ANOVA were significantly different for RDI and LSI, implying that they had varying degrees of resistance to *Foc* race 1. Genotypes that were significantly different from the susceptible controls (Kayinja and Sukali Ndiizi) were subsequently grouped into the highly resistant and the resistant classes by disease severity index (DSI) scores for rhizome discoloration (RDI). Ten out of 18 (~ 55.6%) of the diploids evaluated were classified as either highly resistant or resistant and the tetraploid (Monyet) was grouped as resistant. The results reported in this study are in agreement with those reported by Uma et al. (2011) and Ribeiro et al. (2017) where the authors reported that diploid banana accessions they assessed were resistant to most of the pests and diseases tested. A study by Kumar et al. (2009) also found a majority (4/7 synthetic diploids and 4/6 diploid parents) of the diploid banana cultivars to be resistant to *Foc* race 1. Genotype Kokopo, a fertile diploid and assumed to be a source of vitamin A because of its orange-fleshed fruit colour, was grouped as susceptible. Kokopo can be utilised for genetic studies for *Foc* race 1 and also as a source of vitamin A for incorporation into the East African highland banana (EAHBs) breeding programmes. The Mchare varieties (Hutishamba, Mraru, Mshale, Njuru, Nshonowa, Kahuti, Mlelembo), which are the most common edible bananas in Tanzania, were all susceptible to *Foc* race 1 in the present study. This may pose a threat to the food security in the region and therefore steps to improve the resistance of Mchare bananas against *Foc* race 1 are highly recommended. Accessions that have been identified as resistant or susceptible in the present study can be used as parents to be integrated into breeding programmes to improve the resistance of dessert, plantain and Mchare bananas to *Foc* race 1 and for studying the mechanisms underlying Fusarium wilt resistance.

The standard error means separation and the LSD between the means for leaf symptom index (LSI) could not significantly differentiate the resistant control Mbwazirume from the susceptible control Kayinja. Some genotypes, such as TMB2X8075-7, Kasaska, Borneo, Mwitu Pemba that had shown lower RDI (non-significant RDI values compared to resistant control Mbwazirume), showed advanced yellowing symptoms (i.e. high LSI value) and could not be clearly differentiated from the susceptible controls. The advanced yellowing of these genotypes may not be associated with Fusarium wilt infection since they had lower RDI values or no rhizome discoloration, but is possibly associated with mineral deficiency and/ or excessive water. Ribeiro et al. (2017) reported that a plant can show external characteristics such as advanced yellowing (high LSI) due to nutritional deficiency and excess water, but internally may not exhibit rhizome discoloration (high RDI). According to Li et al. (2015), *Foc* can cause internal corm discoloration without causing any external symptoms such as yellowing of leaves. Ribeiro et al. (2017) and Li et al. (2015) suggested that dissecting the rhizome to verify the absence/presence of discoloration is the most precise evaluation of Fusarium wilt. Therefore, we grouped the germplasm into resistance groups and selected parents contrasting for *Foc* race 1 resistance, only based on the DSI for RDI.

We selected Monyet (*Foc* race 1 resistant) and Kokopo (susceptible) as suitable parents for developing a segregating F_1_ population for assessing the genetic basis of resistance to *Foc* race 1. The tetraploid Monyet was selected to be used as the female because of its moderate female fertility, while Kokopo, a diploid was used as the source of pollen.

The segregation ratios of resistant vs susceptible for the F_1_ progenies fit 13:3 (χ^2^ = 0.12, *P* = 0.73) for RDI and 11:5 (χ^2^ = 3.04, *P*  = 0.08) for PSS. Both 13:3 and 11:5 ratios obtained in current study are a deviation from the expected 9:3:3:1 dihybrid ratio, suggesting that *Foc* race 1 is under the genetic control of at least two dominant genes with epistatic interactions. Previous studies have reported Fusarium wilt to be under the genetic control of a single dominant and single recessive gene. Single dominant genetic controls of Fusarium wilt reports include Larter (1947) who reported that *Foc* race1 was controlled by a single dominant gene in a study of tetraploid progenies obtained by the cross of Gros Michel with a diploid accession. Vakili (1965) also reported *Foc* race1 to be under the control of a single dominant gene in a banana population developed using a homozygous banana parent ‘Pisang Lilin’ as the source of resistance. Fraser-Smith et al. (2016) reported *Foc* subtropical race 4 (SR4) and TR4 to be under the genetic control of a single dominant gene in an F_1_ progeny of self-fertilized *malaccensis* plants. Control of resistance by a recessive gene has also been observed by Ssali et al. (2013) who reported that *Foc* race 1 inheritance was controlled by a single recessive gene in an F_2_ population derived from crosses of ‘Sukali Ndizi’ (AAB) and a resistant diploid banana ‘TMB2X8075′ (AA).

The two dominant genes with epistasis obtained in the current study differing from the most reported single dominant gene model, could be because an early F_1_ generation was used. Ssali et al. (2013) reported that there is more genetic variation in F_2_ banana individuals, which provides a better platform to study mode of inheritance compared to the low variation in F_1_ individuals. Another cause of discrepancy in the gene ratio could be due to the low number of F_1_ progenies used in the current study. Ideal mapping populations should consist of a minimum of 50–250 individuals (Collard et al. 2005). Ortiz and Vuylsteke (1994) attributed the inconclusiveness between the one or two genes model controlling the inheritance of albinism in *Musa* spp*.* to the small sample sizes of below 65 genotypes (a problem inherent in the low reproductive fertility of cultivated parthenocarpic Musa). Other factors that could have affected the genetic ratios in the current study are, the use of single environments for evaluations and heterozygosity between parents. Kammili and Raoof (2014) attributed the different inheritance patterns (15:1, 9:7 and 13:3) of *Fusarium oxysporum* f.sp. *ricini* observed in castor (*Ricinus communis* L.) to the use of a single location for the evaluations and high levels of heterozygosity and heterogeneity within parents used in their study. Therefore, another study is recommended to confirm the genes controlling *Foc* race 1 using an advanced F_2_ population with a large number of progenies. Also, it will be important to confirm the nature of inheritance of resistance to *Foc* race 1 using molecular markers. However, there are no available molecular markers associated with *Foc* race 1 in bananas.

The generation of an F_1_ population segregating for *Foc* race 1 resistance, provided an opportunity to determine heritability of resistance to this trait for the first time in *Musa* spp. We found heritability of 27.8% for RDI, 14.7% for PSS and 13.9% for LSI which can be considered low based on the heritability scale described by Johnson et al. (1955), where heritability of 0–30% is classified as low. Several studies have reported low heritabilities for various pests and diseases in *Musa* spp. Ssali et al. (2016) reported a low heritability of 24.4% for youngest leaf spotted when studying black Sigatoka in secondary triploid banana ‘Matooke’ (*Musa* sp., AAA-EA) hybrids. Arinaitwe et al. (2015) reported a low heritability of 24.0% for total corm damage caused by weevils in an F_2_ diploid population segregating for weevil resistance. Kiggundu (2000) found a low weevil cross section damage heritabilities of 29% for both upper inner and lower outer damage among hybrids of *Musa* spp*.* The low heritability values obtained in the current study suggests that environmental factors play a big role in inheritance of resistance to *Foc* race 1 and therefore, selection based on phenotype is not recommended. Dutta et al. (2013) and Bushra et al. (2017) reported that selection based on phenotype performance is more effective when the heritability estimates are significantly high. It is, therefore, commendable to use molecular markers when selecting for pest and disease resistances in *Musa* spp. because they are not affected by the environment.

## Conclusion

There was high degree of variability among the parental banana germplasm evaluated for *Foc* race 1, indicating that by hybridizing among them, genetic advance would be achieved for resistance to *Foc* race 1. Therefore, the genotypes evaluated are recommended for integration in the banana breeding program for *Foc* race 1 resistance breeding. Resistance to *Foc* race 1 among F_1_ progenies evaluated, was controlled by at least two dominant genes with epistatic interaction. Low heritability of resistance to *Foc* race 1 was observed in the present study, indicating that the expression of this trait was strongly influenced by the environment. Hence, direct phenotypic-based selection for *Foc* race 1 would probably be ineffective and there is a need for marker assisted selection. There is also a need to develop molecular markers for *Foc* race 1 in bananas by identifying *Foc* race 1 quantitative trait loci (QTLs) from the current developed banana population.
